# Supplementary material for: Locality and entanglement of indistinguishable particles
Source: Sci Rep. 2021 Jul 29;11:15478. doi: 10.1038/s41598-021-94991-y (PMC8322085; doi:10.1038/s41598-021-94991-y)
Supplement: Supplementary file 1 — Supplementary Information. [file 41598_2021_94991_MOESM1_ESM.pdf]

Supplementary Information of
"Locality and entanglement of indistinguishable particles"

Till Jonas Frederick Johann[1,2], Ugo Marzolino[2,3]
[1]Ruprecht-Karls-Universität Heidelberg, Germany
[2]Istituto Nazionale di Fisica Nucleare, Sezione di Trieste, Italy
[*]ugo.marzolino@ts.infn.it

June 23, 2021

Entanglement-I

Definition 1 of the main text results from the general definition developed in several approaches [1, 2, 3]. For the sake of completeness, we find conditions for the commutativity of two operators that do not generate entanglement-I. Note that we have not used commutativity in our theorems, although it plays a crucial role the algebraic formulation of entanglement and in the definition of locality [4, 5, 6, 7]. Then, we prove a variant of Theorem 2 of the main text exploiting the commutativity of the operator subsets. These proofs are derived in first quantisation, but they can be recast in second quantisation, e.g., by mapping the basis (3) into the basis (4) of the main text.

The matrix in the larger space $\mathbb{C}^2 \otimes \mathbb{C}^2$ is block diagonal $O \otimes O = \mathsf{S}\big(O \otimes O\big)\mathsf{S} + \mathsf{A}\big(O \otimes O\big)\mathsf{A}$, where S (A) is the projector onto the (anti-)symmetric subspace. Equation (9) of the main text is the matrix representation of the symmetric block, and the antisymmetric block consists of a 1×1 matrix $a_{00}a_{11} - a_{01}a_{10}$. Therefore, commutativity between matrices on the symmetric subspace is equivalent to commutativity between the larger matrices $O \otimes O$.

Lemma 1. *Consider two operators $A = O \otimes O$ and $B = Q \otimes Q$, with Pauli expansions $O = \sum_{\alpha=0}^{3} x_\alpha \sigma_\alpha$, and $Q = \sum_{\alpha=0}^{3} y_\alpha \sigma_\alpha$, and define $\vec{x} = (x_1, x_2, x_3)$ and $\vec{y} = (y_1, y_2, y_3)$. Commutativity $[A, B] = 0$ implies either $x_0 y_0 + \vec{x} \cdot \vec{y} = 0$ or $\vec{x} \wedge \vec{y} = 0$.*

Proof. The commutator $[A, B]$ must be identically zero, and so must be each coefficient in the expansion in Pauli matrices, namely $c_{\epsilon,\eta} = \mathrm{Tr}\big([A, B]\sigma_\epsilon \otimes \sigma_\eta\big)$ $(\epsilon, \eta \in \{0, 1, 2, 3\})$. In particular, if $\epsilon \in \{1, 2, 3\}$ and $\eta = 0$, $c_{\epsilon,\eta} = s z_\epsilon$, with $s = x_0 y_0 + \vec{x} \cdot \vec{y}$ and $z_{1,2,3}$ are components of the external product $\vec{z} = \vec{x} \wedge \vec{y}$. Therefore, either $s = 0$ or $\vec{z} = 0$ is a necessary condition for $[A, B] = 0$. □

Theorem 1. *Given two commuting subsets $\mathcal{A}$ and $\mathcal{B}$ of operators that leave SEP_I invariant, the factorisation condition (2) and Definition (1) of the main text imply either $\mathcal{A}$ or $\mathcal{B}$ consists only of operators propotional to the identity.*

Proof. The commuting subsets $\mathcal{A}$ and $\mathcal{B}$ are formed by operators, A and B respectively, as in Lemma 1. Furthermore, separable-I states are $|\Psi\rangle = |\psi\rangle \otimes |\psi\rangle$, and the factorisation condition (2) in the main text is equivalent to

$$\langle\psi|\psi\rangle^2 \langle\psi|OQ|\psi\rangle^2 = \langle\psi|O|\psi\rangle^2 \langle\psi|Q|\psi\rangle^2, \tag{1}$$

Let us now consider the two conditions imposed by Lemma 1: $s = x_0 y_0 + \vec{x} \cdot \vec{y} = 0$ and $\vec{z} = \vec{x} \wedge \vec{y} = 0$. If $s = 0$,

$$OQ = x_0 \vec{y} \cdot \vec{\sigma} + y_0 \vec{x} \cdot \vec{\sigma} + i \vec{z} \cdot \vec{\sigma}, \quad \vec{\sigma} = (\sigma_1, \sigma_2, \sigma_3). \tag{2}$$

If $|\psi\rangle$ is an eigenvector of $\vec{z} \cdot \vec{\sigma}$, then

$$\langle\psi|\psi\rangle^2 \langle\psi|OQ|\psi\rangle^2 = -\langle\psi|\psi\rangle^4 |\vec{z}|^2 \leqslant 0, \tag{3}$$

while, due to the hermiticity of O and Q,

$$\langle\psi|O|\psi\rangle^2 \langle\psi|Q|\psi\rangle^2 \geqslant 0. \tag{4}$$

Therefore, the factorisation condition (1) is fulfilled only if $\vec{z} = 0$.

Consider now the case $\vec{z} = 0$, which implies $\vec{y} = \gamma \vec{x}$. The factorisation condition (1) is a forth order polynomial in $\langle\psi|\vec{x} \cdot \vec{\sigma}|\psi\rangle$. Furthermore, $\langle\psi|\vec{x} \cdot \vec{\sigma}|\psi\rangle$ spans the real axis when $|\psi\rangle$ varies: recall that we have relaxed the normalisation condition for states. Therefore, the coefficient of all powers of $\langle\psi|\vec{x} \cdot \vec{\sigma}|\psi\rangle$ must vanish. In particular, the coefficient of $\langle\psi|\vec{x} \cdot \vec{\sigma}|\psi\rangle^4$ is γ^2. In conclusion, $\gamma = 0$ and thus one of the subset, either $\mathcal{A}$ and $\mathcal{B}$, is made only of operators proportional to the identity. □

Examples

In this section, we provide some examples of the properties proved in the context of entanglement-I. Consider a generic separable-I state $|\Psi\rangle = |\psi\rangle \otimes |\psi\rangle$, with $|\psi\rangle = c_0|0\rangle + c_1|1\rangle$ as in Definition 1 of the main text, and impose the normalisation $\langle\psi|\psi\rangle = |c_0|^2 + |c_1|^2 = 1$. First of all, the action of single-particle operators $O \otimes \mathbb{1}$ do not preserve the particle permutation symmetry. Indeed, the resulting state is not invariant under the exchange of particles:

$$\big(O \otimes \mathbb{1}\big)|\psi\rangle \otimes |\psi\rangle = \big(O|\psi\rangle\big) \otimes |\psi\rangle. \tag{5}$$

On the other hand, symmetrised single-particle operators $O \otimes \mathbb{1} + \mathbb{1} \otimes O$ generate entanglement-I:

$$\big(O \otimes \mathbb{1} + \mathbb{1} \otimes O\big)\psi\rangle \otimes |\psi\rangle = \big(O|\psi\rangle\big) \otimes |\psi\rangle + |\psi\rangle \otimes \big(O|\psi\rangle\big). \tag{6}$$

The state (6) is not separable-I. If, e.g., either $c_0 = 1$ or $c_1 = 1$ and $O = \sigma_1$ is the first Pauli matrix, the state (6) is $|0\rangle \otimes |1\rangle + |1\rangle \otimes |0\rangle$.

As examples of commuting operators that do not generate entanglement-I, consider $O \otimes O$ and $Q \otimes Q$, with Pauli matrices $O = \sigma_1$ and $Q = \sigma_2$. Therefore,

$$\langle\Psi|\big(O \otimes O\big)\big(Q \otimes Q\big)|\Psi\rangle = -\langle\psi|\sigma_3|\psi\rangle^2 = -\big(|c_0|^2 - |c_1|^2\big)^2, \tag{7}$$

while

$$\langle\Psi|\big(O \otimes O\big)|\Psi\rangle = \langle\psi|\sigma_1|\psi\rangle^2 = 4\big(\mathrm{Re}(\overline{c_0} c_1)\big)^2, \tag{8}$$

$$\langle\Psi|\big(Q \otimes Q\big)|\Psi\rangle = \langle\psi|\sigma_2|\psi\rangle^2 = 4\big(\mathrm{Im}(\overline{c_0} c_1)\big)^2. \tag{9}$$

The factorisation condition is violated,

$$\langle\Psi|\big(O \otimes O\big)\big(Q \otimes Q\big)|\Psi\rangle \neq \langle\Psi|\big(O \otimes O\big)|\Psi\rangle\langle\Psi|\big(Q \otimes Q\big)|\Psi\rangle. \tag{10}$$

For instance, if either $c_0 = 1$ or $c_1 = 1$ the inequality (10) reads $-1 \neq 0$, and if $c_0 = 1/\sqrt{5}, c_1 = 2/\sqrt{5}$ we obtain $-\frac{9}{25} \neq 0$.

Entanglement-II

Definition 2 of the main text follows from the general frameworks in Refs. [8, 9, 1, 2, 10, 3, 11, 12, 13]. It is worthwhile to stress that separable-I states are also separable-II, but there are separable-II states that are entangled-I. Theorem 4 of the main text shows the incompatibility of entanglement-II with the locality notion, exploiting similar results for entanglement-I. Now, we provide a more detailed argument that relies on the explicit forms of operators that leave $\mathsf{SEP}_{\mathsf{II}}$ invariant.

Theorem 2. *Any operator that leaves* $\mathsf{SEP}_{\mathsf{II}}$ *invariant either leaves* $\mathsf{SEP}_{\mathsf{II}} \setminus \mathsf{SEP}_{\mathsf{I}}$ *invariant or sends* $\mathsf{SEP}_{\mathsf{II}}$ *in* $\mathsf{SEP}_{\mathsf{I}}$*.*

Proof. Consider an operator A that leaves $\mathsf{SEP}_{\mathsf{II}}$ invariant, and a state $|\Psi\rangle \in \mathsf{SEP}_{\mathsf{II}} \setminus \mathsf{SEP}_{\mathsf{I}}$. Either $A|\Psi\rangle \in \mathsf{SEP}_{\mathsf{I}}$ or $A|\Psi\rangle \in \mathsf{SEP}_{\mathsf{II}} \setminus \mathsf{SEP}_{\mathsf{I}}$. A necessary and sufficient condition for $A|\Psi\rangle \in \mathsf{SEP}_{\mathsf{I}}$ is equation (8) of the main text, as in the proof of Theorem 3 of the main text,

$$P_A(c_0, \overline{c_0}, c_1, \overline{c_1}) := \langle \Phi_2|A|\Psi\rangle^2 - 2\,\langle \Phi_0|A|\Psi\rangle\langle \Phi_1|A|\Psi\rangle = 0. \tag{11}$$

The crucial difference with the case $|\Psi\rangle \in \mathsf{SEP}_{\mathsf{I}}$ of Theorem 3 of the main text is that the polynomial P_A now depends also on complex conjugates $\overline{c_0}$ and $\overline{c_1}$. A necessary and sufficient condition for $A|\Psi\rangle \in \mathsf{SEP}_{\mathsf{II}} \setminus \mathsf{SEP}_{\mathsf{I}}$ is equation (22) of the main text, exactly as in the proof of Theorem 3 of the main text.

Now, also equation (11) can have no solutions. Therefore, fixing c_0 and $\operatorname{Im} c_1$, each of equation (11) and equation (22) of the main text either have a finite number (possibly zero) of solutions or are tautologies. As in the proof of Theorem 3 of the main text, if both equations have finitely many solutions, there are states in $\mathsf{SEP}_{\mathsf{II}}$ that are send out of $\mathsf{SEP}_{\mathsf{II}}$, contradicting the hypothesis. Therefore, one equation is a tautology. For the other equation there are two possibilities: it has no solutions otherwise there are states sent to both $\mathsf{SEP}_{\mathsf{I}}$ and $\mathsf{SEP}_{\mathsf{II}} \setminus \mathsf{SEP}_{\mathsf{I}}$ contradicting the linearity of A, or A annihilates any solution $|\Psi\rangle$.

If equation (11) is the tautology, then $A \cdot \big(\mathsf{SEP}_{\mathsf{II}} \setminus \mathsf{SEP}_{\mathsf{I}}\big) \subset \mathsf{SEP}_{\mathsf{I}}$ which implies, together with Theorem 3 of the main text, $A \cdot \mathsf{SEP}_{\mathsf{II}} \subset \mathsf{SEP}_{\mathsf{I}}$. If equation (22) of the main text is the tautology, then $A \cdot \big(\mathsf{SEP}_{\mathsf{II}} \setminus \mathsf{SEP}_{\mathsf{I}}\big) \subset \mathsf{SEP}_{\mathsf{II}} \setminus \mathsf{SEP}_{\mathsf{I}}$. □

Theorem 3. *The operators that leave* $\mathsf{SEP}_{\mathsf{II}}$ *invariant are represented on* $\mathbb{C}^2 \otimes \mathbb{C}^2$ *as* $O \otimes O$ *where* O *is proportional to a unitary matrix.*

Proof. From Theorem 3 of the main text, the operators considered here leave $\mathsf{SEP}_{\mathsf{I}}$ invariant, and are therefore represented as $O \otimes O$ on the larger Hilbert space $\mathbb{C}^2 \otimes \mathbb{C}^2$, by Theorem 1 of the main text. Consider a separable-II state

$$|\Psi\rangle = \mathsf{S}\,|\psi\rangle \otimes |\psi^{\perp}\rangle \in \mathsf{SEP}_{\mathsf{II}} \setminus \mathsf{SEP}_{\mathsf{I}}, \tag{12}$$

with $\langle \psi|\psi^{\perp}\rangle = 0$.

If $O \otimes O$ sends $|\Psi\rangle$ to $\mathsf{SEP}_{\mathsf{I}}$, namely

$$O \otimes O|\Psi\rangle = \mathsf{S}\big(O|\psi\rangle \otimes O|\psi^{\perp}\rangle\big) \in \mathsf{SEP}_{\mathsf{I}}, \tag{13}$$

then $O|\psi\rangle = O|\psi^{\perp}\rangle$ which implies $O = \lambda|+\rangle\langle+|$ with $|+\rangle = |\psi\rangle + |\psi^{\perp}\rangle$. This result must hold for any basis $\big\{|\psi\rangle, |\psi^{\perp}\rangle\big\}$, according to Theorem 2. Therefore, λ must be zero, and there are no operators that transform $\mathsf{SEP}_{\mathsf{II}} \setminus \mathsf{SEP}_{\mathsf{I}}$ to $\mathsf{SEP}_{\mathsf{I}}$.

The other possibility left by Theorem 2 is that

$$O \otimes O|\Psi\rangle = \mathsf{S}\big(O|\psi\rangle \otimes O|\psi^{\perp}\rangle\big) \in \mathsf{SEP}_{\mathsf{II}} \setminus \mathsf{SEP}_{\mathsf{I}} \tag{14}$$

for any basis $\big\{|\psi\rangle, |\psi^{\perp}\rangle\big\}$. From the Definition 2 of the main text,

$$\langle\psi|O^\dagger O|\psi^\perp\rangle = 0, \qquad \forall\,\big\{|\psi\rangle, |\psi^\perp\rangle\big\}. \tag{15}$$

The latter equation implies that $O^\dagger O$ is diagonal in any basis, thus it must be proportional to the identity matrix. Therefore, O is proportional to a unitary matrix. □

Corollary 1. *The operators that leave* $\mathsf{SEP}_{\mathsf{II}}$ *invariant do not form an algebra.*

Proof. If $O\otimes O$ is in the algebra, also $O^\dagger O\otimes O^\dagger O \propto \mathbb{1}$ and thus the identity are in the algebra. Nevertheless, linear combinations of $O\otimes O$ and the identity are not of the form required by Theorem 3. □

In conclusion, it is not possible to identity an algebra of operators that do not generate entanglement-II, and this notion of entanglement is not compatible with the existence of local subsystems defined by subalgebras of their local operators.

Examples

In this section, we discuss some examples of the above theorems for entanglement-II. We remind that separable-I states are also separable-II (see Definition 2 in the main text), and that operators that do not generate entanglement-II are special cases of those that do not gneerate entanglement-I (see Theorem 3). In particular, the examples discussed for entanglement-I applies also to the framework of entanglement-II. The only difference is that it is not enough to compare states (6), that result from the action of symmetrised single-particle operators $O\otimes\mathbb{1}+\mathbb{1}\otimes O$, with separable-I states, but rather with the larger class of separable-II states. Nevertheless, the condition that the state (6) is separable-II, although not separable-I, reads $\langle\psi|O|\psi\rangle = 0$ (see Definition 2 in the main text). This condition, together with the arbitrariness of the state $|\psi\rangle$, implies that O is the zero operator. Therefore, symmetrised single-particle operators $O\otimes\mathbb{1}+\mathbb{1}\otimes O$ generate entanglement-II.

We now complement the examples in the previous section with others that exploit separable-II states that are not separable-I, namely

$$|\Psi\rangle = \frac{1}{\sqrt{2}}\big(|\psi\rangle\otimes|\psi^\perp\rangle + |\psi^\perp\rangle\otimes|\psi\rangle\big), \tag{16}$$

where

$$|\psi\rangle = c_0|0\rangle + c_1|1\rangle, \tag{17}$$

$$|\psi^\perp\rangle = \overline{c_1}|0\rangle - \overline{c_0}|1\rangle, \tag{18}$$

with the normalisation condition $|c_0|^2+|c_1|^2=1$. Consider, as in section , the operators $O\otimes O$ and $Q\otimes Q$ with $O=\sigma_1$ and $Q=\sigma_2$, that do not generate entanglement-II. The expectation values of these operators are

$$\langle\Psi|\big(O\otimes O\big)\big(Q\otimes Q\big)|\Psi\rangle = -\langle\psi|\sigma_3|\psi\rangle\langle\psi^\perp|\sigma_3|\psi^\perp\rangle - \big|\langle\psi^\perp|\sigma_3|\psi\rangle\big|^2 = |c_0|^4+|c_1|^4-6\,|c_0\,c_1|^2, \tag{19}$$

and

$$\langle\Psi|\big(O\otimes O\big)|\Psi\rangle = \big(\langle\psi|\sigma_1|\psi\rangle\langle\psi^\perp|\sigma_1|\psi^\perp\rangle + \big|\langle\psi^\perp|\sigma_1|\psi\rangle\big|^2\big) = \Big(\big|c_1^2-c_0^2\big|^2 - 4\big(\mathrm{Re}(\overline{c_0}c_1)\big)^2\Big), \tag{20}$$

$$\langle\Psi|\big(Q\otimes Q\big)|\Psi\rangle = \big(\langle\psi|\sigma_2|\psi\rangle\langle\psi^\perp|\sigma_2|\psi^\perp\rangle + \big|\langle\psi^\perp|\sigma_2|\psi\rangle\big|^2\big) = \Big(\big|c_0^2+c_1^2\big|^2 - 4\big(\mathrm{Im}(\overline{c_0}c_1)\big)^2\Big). \tag{21}$$

Therefore, the factorisation condition (2) in the main text is violated in general, as in equation (10).

Entanglement-III

Note that Definition 3 of the main text is called entanglement-IV in [6] whereas entanglement-III therein is the definition called SSR-entanglement in the main text.

In order to define separable-III states in Definition 3 of the main text, we need to introduce some preliminary notions. First of all, define reductions from the two-particle Hilbert space to the single-particle Hilbert space.

$$\Pi_\psi\, \mathsf{S}\, |\phi\rangle \otimes |\zeta\rangle = \langle\psi|\phi\rangle|\zeta\rangle + \eta\langle\psi|\zeta\rangle|\phi\rangle \ , \tag{22}$$

where $\eta = +1(-1)$ for bosons (fermions). Within this approach, a so-called reduced single-particle density matrix is defined relative to a single-particle subspace $\mathcal{K}$:

$$\rho^{(1)} = \frac{1}{\sum\limits_{\substack{k:\{|\psi_k\rangle\}_k \\ \text{ONB of } \mathcal{K}}} \big|\big|\Pi_{\psi_k}|\Psi\rangle\big|\big|^2} \sum_{\substack{k:\{|\psi_k\rangle\}_k \\ \text{ONB of } \mathcal{K}}} \Pi_{\psi_k}|\Psi\rangle\langle\Psi|\Pi^\dagger_{\psi_k}, \tag{23}$$

where $\big\{|\psi_k\rangle\big\}_k$ is any orthonormal basis (ONB) of $\mathcal{K}$. In second quantisation, $\rho^{(1)}$ reads

$$\rho^{(1)} = \frac{1}{\sum\limits_{\substack{k:\{|\psi_k\rangle\}_k \\ \text{ONB of } \mathcal{K}}} \big|\big|\mathfrak{a}_{\psi_k}|\Psi\rangle\big|\big|^2} \sum_{\substack{k:\{|\psi_k\rangle\}_k \\ \text{ONB of } \mathcal{K}}} \mathfrak{a}_{\psi_k}|\Psi\rangle\langle\Psi|\mathfrak{a}^\dagger_{\psi_k}, \tag{24}$$

where $\mathfrak{a}^\dagger_{\psi_k}$ ($\mathfrak{a}_{\psi_k}$) creates (annihilates) a particle in the state $|\psi_k\rangle$, and $\big[\mathfrak{a}_{\psi_k}, \mathfrak{a}^\dagger_{\psi_{k'}}\big] = \delta_{k,k'}$. Note that the density matrix $\rho^{(1)}$ does not reproduce expectations of single-particle observables [14], and, yet, is used to define entanglement.

A two-particle state $|\Psi\rangle$ is said to be separable-III if $\rho^{(1)}$ is a one-dimensional projector, i.e. if $\big(\rho^{(1)}\big)^2 = \rho^{(1)}$. Since $\mathcal{K} = \mathrm{span}\big\{|L,\sigma\rangle\big\}_\sigma$, the states $|\psi_k\rangle$ in equation (23) are of the form $|L,\sigma\rangle$ for some internal state σ. The condition $\big(\rho^{(1)}\big)^2 = \rho^{(1)}$ implies that $\rho^{(1)}$ has only one non zero eigenvalue, and so there exists a basis $\big\{|L,\sigma\rangle, |L,\sigma^\perp\rangle\big\}$, with $\langle\sigma|\sigma^\perp\rangle = 0$, such that $\Pi_{L,\sigma^\perp}|\Psi\rangle = 0$. Therefore, separable-III states can be expressed with single-particle states with components along the states $|L,\sigma\rangle$, $|R,\sigma\rangle$, $|R,\sigma^\perp\rangle$, but not along $|L,\sigma^\perp\rangle$. Definition 3 of the main text follows from the above considerations.

Examples

In this section, we present some examples for the impossibility to reconcile the definition of entanglement-III with the Werner's formulation. First of all, consider separable-III states with two particles in the left location, namely separable-III states that lie in the support of the projector P_{LL} defined in equations (25) of the main text. The support of P_{LL} is spanned by states where only internal degrees of freedom varies. Therefore, this subspace is isomorphic to $\mathbb{C}^2 \otimes \mathbb{C}^2$, and entanglement-III in this subspace is the same as entanglement-I. Therefore, all the examples shown in section applies also here.

Consider now more general examples of separable-III states:

$$|\Psi\rangle = c_{LL}|L,0\rangle^{\otimes 2} + c_{LR}\,\mathsf{S}\,|L,0\rangle \otimes |R,1\rangle + c_{RR}|R,1\rangle^{\otimes 2}, \tag{25}$$

in first quantization, or

$$|\Psi\rangle = c_{LL}\,\frac{\big(\mathfrak{a}^\dagger_{L,0}\big)^2}{\sqrt{2}}|\mathrm{vac}\rangle + c_{LR}\,\mathfrak{a}^\dagger_{L,0}\mathfrak{a}^\dagger_{R,1}|\mathrm{vac}\rangle + c_{RR}\,\frac{\big(\mathfrak{a}^\dagger_{R,1}\big)^2}{\sqrt{2}}|\mathrm{vac}\rangle, \tag{26}$$

in second quantization, with the normalisation $\langle\Psi|\Psi\rangle = |c_{LL}|^2 + |c_{LR}|^2 + |c_{RR}|^2 = 1$. Note that thess state are separable-III for both choices of the single-particle subspace $\mathcal{K} = \mathrm{span}\{|L,0\rangle, |L,1\rangle\}$ and $\mathcal{K} = \mathrm{span}\{|R,0\rangle, |R,1\rangle\}$ (see the comments before Definition 3 in the main text and equations (23) and (24)). Consider also the operators

$$A = |L\rangle\langle L| \otimes \sigma_3 \otimes \mathbb{1} + \mathbb{1} \otimes |L\rangle\langle L| \otimes \sigma_3, \tag{27}$$

$$B = |R\rangle\langle R| \otimes \sigma_3 \otimes \mathbb{1} + \mathbb{1} \otimes |R\rangle\langle R| \otimes \sigma_3, \tag{28}$$

where $\mathbb{1}$ is the identity matrix on the single-particle Hilbert space with spatial and internal degrees of freedom. These operators can be written in second quantization as

$$A = a^\dagger_{L,0} a_{L,0} - a^\dagger_{L,1} a_{L,1}, \tag{29}$$

$$B = a^\dagger_{R,0} a_{R,0} - a^\dagger_{R,1} a_{R,1}, \tag{30}$$

and are considered local within the theory of entanglement-III [15, 16]. Expectation values are

$$\langle\Psi|AB|\Psi\rangle = -|c_{LR}|^2, \tag{31}$$

$$\langle\Psi|A|\Psi\rangle = 2\,|c_{LL}|^2 + |c_{LR}|^2, \tag{32}$$

$$\langle\Psi|B|\Psi\rangle = -2\,|c_{RR}|^2 - |c_{LR}|^2. \tag{33}$$

Therefore, the factorisation condition is violated:

$$\langle\Psi|AB|\Psi\rangle \neq \langle\Psi|A|\Psi\rangle\langle\Psi|B|\Psi\rangle. \tag{34}$$

For instance, if $c_{LR} = 0$ inequality (34) reads $0 \neq -4|c_{LL}|^2|c_{RR}|^2$, and if $c_{RR} = 0$ we obtain $1 \neq 1 + |c_{LL}|^2$. The factorisation condition is fulfilled only if $c_{LL} = c_{RR} = 0$, and thus $c_{LR} = 1$ which implies that the states $|\Psi\rangle$ lie in the support of the projector P_{LR}. Within this subspace, entanglement-III is equivalent to SSR-entanglement, studied in [17, 18, 19] (see also the section Discussions in the main text), that is compatible with the Werner's formulation and is a special case of mode-entanglement [6].

References

[1] Paskauskas, R. & You, L. Quantum correlations in two-boson wave functions. *Phys. Rev. A* **64**, 042310 (2001).

[2] Eckert, K., Schliemann, J., Bruß, D. & Lewenstein, M. Quantum correlations in systems of indistinguishable particles. *Ann. Phys.* **299**, 88 (2002).

[3] Grabowski, J., Marek Kuŝ, M. & Marmo, G. Entanglement for multipartite systems of indistinguishable particles. *J. Phys. A* **44**, 175302 (2011).

[4] Werner, R. F. Quantum states with einstein-podolsky-rosen correlations admitting a hidden-variable model. *Phys. Rev. A* **40**, 4277 (1989).

[5] Chitambar, E., Leung, D., Mancinska, L., Ozols, M. & Winter, A. Everything You Always Wanted to Know About LOCC (But Were Afraid to Ask). *Comm. Math, Phys.* **328**, 303 (2014).

[6] Benatti, F., Floreanini, R., Franchini, F. & Marzolino, U. Entanglement in indistinguishable particle systems. *Phys. Rep.* **878**, 1 (2020).

[7] Sengupta, K., Zibakhsh, R., Chitambar, E. & Gour, G. Quantum Bell Nonlocality is Entanglement (2020). Preprint arXiv:2012.06918.

[8] Herbut, F. How to distinguish identical particles. *Am. J. Phys.* **69**, 207 (2001).

[9] Schliemann, J., Cirac, J. I., Kuś, M., Lewenstein, M. & Loss, D. Quantum correlations in two-fermion systems. *Phys. Rev. A* **64**, 022303 (2001).

[10] Plastino, A. R., Manzano, D. & Dehesa, J. S. Separability criteria and entanglement measures for pure states of N identical fermions. *EPL* **86**, 20005 (2009).

[11] Li, Y. S., Zeng, B., Liu, X. S. & Long, G. L. Entanglement in a two-identical-particle system. *Phys. Rev. A* **64**, 054302 (2001).

[12] Ghirardi, G., Marinatto, L. & Weber, T. Entanglement and Properties of Composite Quantum Systems: a Conceptual and Mathematical Analysis. *J. Stat. Phys.* **108**, 49 (2002).

[13] Iemini, F. & Vianna, R. O. Computable measures for the entanglement of indistinguishable particles. *Phys. Rev. A* **87**, 022327 (2013).

[14] Benatti, F., Floreanini, R., Franchini, F. & Marzolino, U. Remarks on entanglement and identical particles. *Open Syst. Inf. Dyn.* **24**, 1740004 (2017).

[15] Lo Franco, R. & Compagno, G. Quantum entanglement of identical particles by standard information-theoretic notions. *Sci. Rep.* **6**, 20603 (2016).

[16] Castellini, A., Bellomo, B., Compagno, G. & Lo Franco, R. Activating remote entanglement in a quantum network by local counting of identical particles. *Phys. Rev. A* **99**, 062322 (2019).

[17] Wiseman, H. M. & Vaccaro, J. A. Entanglement of Indistinguishable Particles Shared between Two Parties. *Phys. Rev. Lett.* **91**, 097902 (2003).

[18] Ichikawa, T., Sasaki, T. & Tsutsui, I. Separability of N-particle fermionic states for arbitrary partitions. *J. Math. Phys.* **51**, 062202 (2010).

[19] Sasaki, T., Ichikawa, T. & Tsutsui, I. Entanglement of indistinguishable particles. *Phys. Rev. A* **83**, 012113 (2011).
